# Optical Polarization-Based Measurement Methods for Characterization of Self-Assembled Peptides’ and Amino Acids’ Micro- and Nanostructures

**DOI:** 10.3390/molecules27061802

**Published:** 2022-03-10

**Authors:** Amir Handelman

**Affiliations:** Department of Electrical Engineering, Faculty of Engineering, Holon Institute of Technology, Holon 5810201, Israel; handelmana@hit.ac.il

**Keywords:** peptides and amino acids, optical polarization, Raman spectroscopy, waveguide, imaging, birefringence

## Abstract

In recent years, self-assembled peptides’ and amino acids’ (SAPA) micro- and nanostructures have gained much research interest. Here, description of how SAPA architectures can be characterized using polarization-based optical measurement methods is provided. The measurement methods discussed include: polarized Raman spectroscopy, polarized imaging microscopy, birefringence imaging, and fluorescence polarization. An example of linear polarized waveguiding in an amino acid Histidine microstructure is discussed. The implementation of a polarization-based measurement method for monitoring peptide self-assembly processes and for deriving molecular orientation of peptides is also described.

## 1. Introduction

The increasing interest in the field of “organic photonics” has accelerated the search for novel optical materials at the nano- and micro-scales [1,2,3]. In organic photonics, optical elements are made of or comprise organic materials to achieve certain improved functionality aspects. One well-known example of devices that use organic photonics is organic light-emitting diode (OLED) displays [2].

One common property of many organic materials is their ability to self-assemble into ordered architectures, a property that may be considered as a natural “bottom-up” fabrication approach. The self-assembly process is reversible and does not require external energy; it is governed by specific interactions or forces between the assembled entities [4]. A self-assembly process occurs in many biological systems, and it relies upon the basic concept of supramolecular chemistry [5], which was first described by the 1987 Nobel Prize laureate Jean-Marie Lehn [6].

Amino acids serve as basic building blocks of peptides and complex biological structures and are also able to self-assemble into various ordered architectures. Decoding the amino acids and their sequences opened the avenue for the development of new materials, which are called “bioinspired” and are based on chemically synthesized peptides [7]. These synthesized peptides are also supramolecular materials, which are capable of self-organization into nanostructures with different shapes and sizes. Theoretical and experimental works have shown that peptide micro- and nanostructures are created by weak, dynamic, and reversible non-covalent interactions [1,8,9].

Fabrication of SAPA nano- and microstructures is performed by dissolving peptide or amino acid powders in different solvents [10] and allowing the resulting solutions to dry. The various SAPA architectures can be obtained by controlling different conditions that affect the self-assembly process, such as altering the amino acid’s sequence [11], protecting the amine group in the peptide’s molecule [12], and changing the solvent properties (e.g., polarity, dielectric constant, pH, etc.) [13], to name but a few. Some examples to such micro-architectures that were obtained from different peptide and amino acid molecules include hollow rectangular nanotubes from β-peptide ACPC4 peptide [14], micro-vesiculars obtained from terminally protected tripeptides Boc–Leu–Aib–Val–m-ABA–OMe, [15], hexagonal hollow diphenylalanine (FF) nanotubes [7], and “necklaces” that result from the co-assembly of FF and its tert-butyl dicarbonate (Boc) protected analogue [16].

Since the discovery of hollow peptide nanotubes by Ghadiri [17] in 1993, which were assembled from bioinspired cyclic peptides based on alternating D- and L-amino acid residues, the research in the field of self-assembled peptide and amino acids (SAPA) micro- and nanostructures has bloomed. Major research efforts in the past 30 years have pointed toward using various SAPA micro- and nanostructures in various electronic, biologic, and optical disciplines [18,19,20,21,22,23,24]. Some noteworthy devices that exploit SAPA micro- and nanostructures are autonomous biochemical motors [25], energy storage devices [26], light-emitting field-effect transistors [27], surgical sutures [28], and supercapacitors [29,30].

One major factor that determines the physical and chemical properties of organic materials, and specifically SAPA micro- and nanostructures, is molecular orientation [31]. The molecular orientation of a material is the angular position and shape of its molecular distribution [32]. In many cases, the molecular distribution can be considered only in respect of the angle between a directional reference and the molecular chain axis. It is important to study the molecular orientation of a material, because this affects its optical, mechanical, and electronic properties. For example, molecular orientation determines light outcoupling in OLED, the charge carrier mobility in organic thin-film transistors [31], and the tensile modulus of polymers [33].

Polarization-based optical measurement methods are very useful in the analysis of the molecular orientations of materials, and, thus, these methods are implemented in numerous material-science studies, including into the characterization of SAPA micro- and nanostructures.

There are many papers and books that review the evolving field of self-assembled peptides and amino acids [18,19,20,21,22,23,24]. However, there is currently no ad hoc review of optical polarization-based measurement methods that are aimed to study SAPA architectures. In this paper, this gap is filled by describing the recent results of the polarization-based characterization of SAPA micro- and nanostructures and also particular optical properties that were recently discovered in a microstructure of the amino acid Histidine. The particular optical properties that were discovered with respect to Histidine show the potential for possible future discoveries that may be achieved using one or more of the above-mentioned polarization-based optical measurement methods in other organic materials’ architectures. The use of a polarization-based measurement method for monitoring peptide self-assembly processes is also described.

It is important to note that there are many methods and tools that have been used to characterize SAPA nano- and microstructures. Different microscopy tools, such as Scanning Electron Microscopy (SEM) and Atomic Force Microscopy (AFM) [34], were used extensively to gain information on the morphology of SAPA micro- and nanostructures. X-ray Diffraction (XRD) and Transmission Electron Microscopy (TEM) [35] were used to decipher the crystallographic structure of various SAPA microstructures. Optical methods, such as Circular Dichroism (CD) and Fourier-transform infrared (FTIR) spectroscopy, were used to obtain the secondary structures of various SAPA micro- and nano-architectures [23]. 

This paper is organized as follows. In Section 2, a brief description of some key concepts in the field of optical polarization is provided. This brief description is provided in order to assist in understanding the principles of polarization-based optical measurements. In Section 3, examples from the scientific literature of various polarization-based measurement methods for the characterization of peptides’ and amino acids’ micro- and nanostructures are provided. In Section 4, the ability of a microstructure of the amino acid Histidine to passively guide linear polarized light is described. Such capability shows the potential for light-guiding applications within or between organic elements. The paper is then concluded in Section 5.

## 2. Key Concepts in Optical Polarization

The polarization of light is one of the most remarkable phenomena in nature and has led to numerous discoveries and applications. The theory of optical polarization is reviewed in numerous books on optics [36,37,38,39]. In this paper, a brief description of some of the key concepts in optical polarization is provided in order to lay the foundation for the next sections.

Polarization describes the direction of the oscillating electric field [36,37,38,39]. The reason the electric field vector, **E**, was chosen to define the state of the polarization of light waves is because the electric field is involved in most light–matter interactions, and, in many media, the refractive index depends on the direction of the electric field.

**Figure 1:**
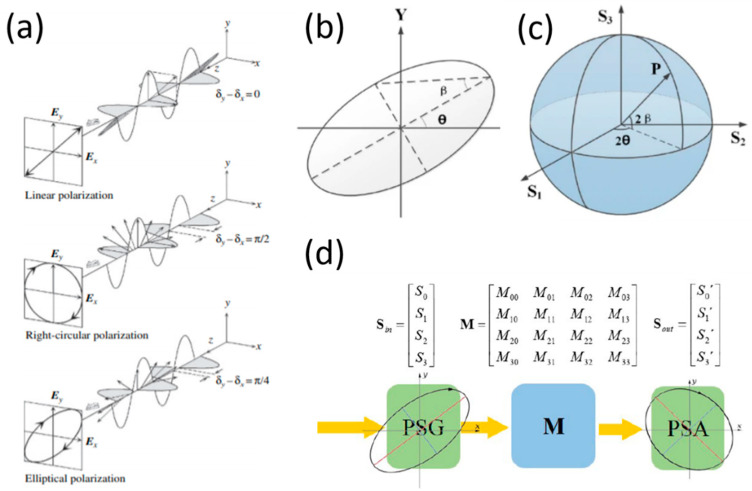
(**a**) Propagation and corresponding trajectory of the electric field vector of linear polarization, right-circular polarization, and elliptical polarization [37], reproduced with permission from H. Fujiwara., *Spectroscopic Ellipsometry: Principles and Applications*, published by John Wiley & Sons, Ltd., 2007; (**b**) representation of the polarization ellipse; (**c**) the Poincaré sphere [40], reproduced with permission from Sun, X., Geng, Y., Zhu, Q. et al., *Sci. Rep.*, published by Springer Nature, 2020; (**d**) Illustration of polarization state generator (PSG) and polarization state analyzer (PSA) used for probing Mueller matrix, reproduced with permission from O. Arteaga and B. Kahr, J. *Opt. Soc. Am. B 36*, F72-F83 (2019), © The Optical Society [42].

There are several combinations of the amplitudes and phases of light waves that lead to two important types of polarization. These combinations, which are known as degenerate polarization states, include linearly horizontal (or vertical) polarized light (LHP/LVP) and right (or left) circularly polarized light (RCP/LCP). Figure 1a shows a representation of linear, circular, and elliptical polarized light, where the phase differences between the electric fields parallel to the *x* and *y* axes are 0, π/2, and π/4, respectively [37].

The state-of-polarization (SOP) may be represented by the polarization ellipse (Figure 1b) or by the Poincaré Sphere [36,38,40] (Figure 1c). The SOP of light is determined by the shape of the polarization ellipse (the direction of the major axis) and its ellipticity (the ratio of the minor axis to the major axis of the ellipse). The size of the polarization ellipse determines the intensity of the electric field. The Poincaré Sphere is a three-dimensional (3D) representation of the polarization ellipse. Any point on the sphere can be expressed in terms of the spherical coordinates of the sphere, i.e., the orientation angle, θ, and the ellipticity angle, β.

The main problem with the orientation and ellipticity angles is that the angles are not directly measurable [36]. In 1852, George Gabriel Stokes laid out a set of four measurable parameters, grouped into a column vector, which were derived by time averaging the polarization ellipse [38]. These parameters are known as the Stokes polarization parameters, and the vector of these parameters is known as the Stokes vector. The Stokes parameters are written as $\;S_{0},S_{1},\;S_{2},\;S_{3}$, and they can be expressed as below [38,41]:

(1) $$S=\left(\begin{array}{c} S_{0}\\ S_{1}\\ \begin{array}{c} S_{2}\\ S_{3} \end{array} \end{array}\right)=\left(\begin{array}{c} {\left|E_{x}\right|}^{2}+{\left|E_{y}\right|}^{2}\\ {\left|E_{x}\right|}^{2}-{\left|E_{y}\right|}^{2}\\ \begin{array}{c} E_{x}^{*}E_{y}+E_{y}^{*}E_{x}\\ i\left(E_{y}^{*}E_{x}-E_{x}^{*}E_{y}\right) \end{array} \end{array}\right)=\left(\begin{array}{c} I_{H}+I_{V}\\ I_{H}-I_{V}\\ \begin{array}{c} I_{45}-I_{-45}\\ I_{R}-I_{L} \end{array} \end{array}\right)=S_{0}\left(\begin{array}{c} 1\\ \cos\left(2\beta \right)\cos\left(2\theta \right)\\ \begin{array}{c} \cos\left(2\beta \right)\sin\left(2\theta \right)\\ \sin\left(2\beta \right) \end{array} \end{array}\right).$$

Here, $I_{H}$ is the intensity measured in the horizontal direction, $I_{V}$ is the intensity measured in the vertical direction, $I_{\pm 45}$ is the intensity measured at ±45°, and $I_{L},\;\mathrm{and}\;I_{R}$ are the intensities measured in the left and right circular polarizations, respectively [41].

When polarized light travels through a polarizing material, a new polarization state is formed at the output of the material. As such, the transformation between the input polarization state (marked as the vector $S_{in}$) and the output polarization state (marked as the vector $S_{out}$) is given by a 4 × 4 matrix known as a Mueller matrix [38,42]. The input polarization is generated by a polarization-state generator (PSG), and the output polarization is determined by a polarization-state analyzer (PSA) [41]. Figure 1d depicts the input-to-output polarization system representation that comprises the PSG, the Mueller matrix M, and the PSA. Knowledge of all 16 Mueller matrix elements provides the full amount of information about the polarized-light–matter interaction, and, therefore, the mathematical analysis and experimental measurement of the Mueller matrix is of great importance.

## 3. Polarization-Based Measurement Methods for Characterization of Peptides and Amino Acids

In this section, a review of common polarization-based optical measurement methods that are used to characterize peptides’ and amino acids’ self-assembled micro- and nanostructures is provided. Most of these methods are aimed at estimating the molecular orientations of various SAPA architectures, but some methods are also used for other applications, as is detailed in the following sections.

### 3.1. Polarized Raman Spectroscopy

Raman spectroscopy [43] is a non-destructive, label-free light-scattering technique that is used to probe light–matter interactions. Specifically, Raman spectroscopy (that relies on inelastic light scattering), is used to analyze the vibrational and rotational molecular modes of a material by monitoring perturbations in the molecular polarizability caused by incident (laser) light [43]. When a polarized laser light is used, information about the molecular orientation can be obtained, and the technique is known as Polarized Raman Spectroscopy [44]. Polarized Raman spectra allow for the extraction of information on the molecular conformation of materials, since the Raman scattering intensity depends on the molecular polarizability tensor [45]. In the following paragraphs, examples of the implementation of polarized Raman spectroscopy for the study of the molecular conformations of SAPA micro- and nanostructures are given.

**Figure 2:**
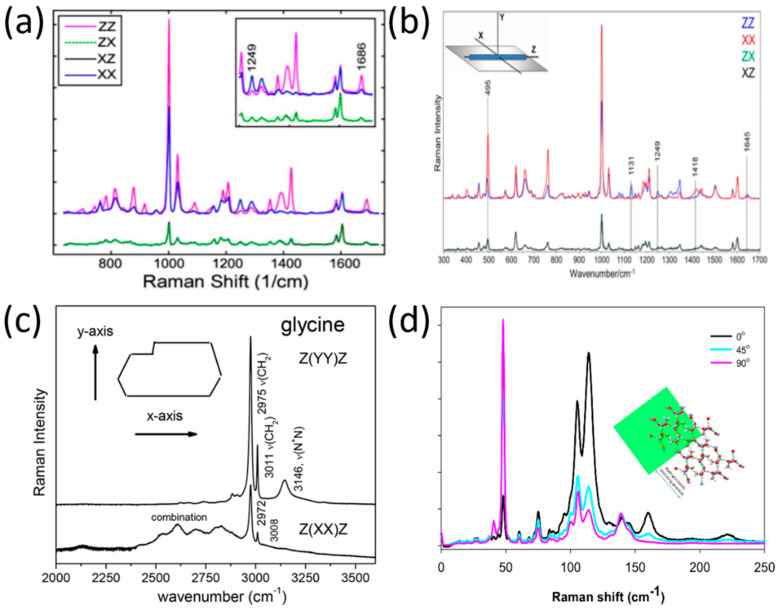
Polarized Raman spectra in peptides and amino acids. (**a**) Raman spectra of L-diphenylalanine di-peptide nanotube, reproduced with permission from B. Lekprasert, V. Sedman, C. J. Roberts, S. J. B. Tedler, I. Notingher, *Opt. Lett*. 35, 4193–4195 (2010) © The Optical Society [46]. (**b**) Raman spectra of di-D-phenylalanine nanotubes [47], reproduced with permission from Igor K. Lednev, Rajesh R. Naik, Milana C. Vasudev, et al., *J. of Raman Spect.*, published by John Wiley and Sons, 2016. (**c**) Polarized Raman spectra of α-form glycine for two scattering geometries [48], reproduced with permission from Paulo T.C. Freire, Felipe M. Barboza, José A. Lima, Francisco E.A. Melo and Josué Mendes Filho, IntechOpen, 2017. (**d**) Polarized Raman spectra of the L-alanine single crystal from the (101) plane in three-beam polarization directions: zero (black), 45 (cyan), and 90 (pink) degrees. The inset shows hydrogen bonds’ simulation relative to the (101) plane [49], reproduced with permission from Nemtsov, I.; Aviv, H.; Mastai, Y.; Tischler, Y.R., *Crystals*, published by MDPI, 2019.

Notingher et al. derived the molecular orientation in the dipeptide L-diphenylalanine (FF) self-assembled nanotubes by applying polarized Raman spectroscopy [46]. As is evident from their findings (presented in Figure 2a), the 1002 cm^−1^ (phenyl group) and the 1686 cm^−1^ (C=O) band exhibited stronger intensities in ZZ rather than in XX configurations (the Z-axis was selected as the direction along the FF nanotubes), while the Raman band at 1249 cm^−1^ (C-N) exhibited maximum intensity in the crossed configuration, XX. As such, the authors concluded that the C=O side chains have parallel alignment with the nanotube axis, and that the C-N backbone vibrations are aligned perpendicular to the nanotube axis.

In a later study by Lednev et al., [47], the researchers used polarized Raman spectroscopy at a larger spectral range (300–1800 cm^−1^) to determine the orientation of di-D-phenylalanine (D-FF) molecules within a nanotube. As evident from Figure 2b, there are differences in several band intensities (1418 cm^−1^, 1131 cm^−1^, 495 cm^−1^) in the XX and ZZ polarizations. The application of polarized Raman spectroscopy allowed the authors of this study to determine the cylindrical symmetry of the nanotube, the orientation of the NH_3_^+^ rocking mode, and the orientation of the COO^−^ group in relation to the nanotube axis.

However, not only was the molecular orientation of peptide structures deduced by polarized Raman spectroscopy, but also the amino acid structures. For example, polarized Raman spectra of the amino acid (α-form) glycine [48] were recorded by Filho et al. for two scattering geometries, as seen in Figure 2c. The implementation of polarized Raman spectroscopy allowed stretching modes that are specific to the glycine polymorphic form to be revealed; these cannot be differentiated without using a polarization technique.

In another study [49], Tischler et al. used low-frequency polarized Raman spectroscopy in order to reveal the molecular orientation of a single organic microcrystal made of the amino acid L-alanine. The Raman spectra at the polarization directions 0°, 45°, and 90° are shown in Figure 2d, relative to the (101) plane. The conclusion from the Raman spectra presented in Figure 2d is that the polarization direction has a major effect on the distribution of photons in both spectral regions of the hydrogen bond stretching modes (parallel beam direction) and shear modes (perpendicular beam direction). These findings allowed the authors to construct a simulation of the hydrogen bond’s network within the single microcrystal.

The works mentioned above are merely examples showing the kind of information that was obtained using polarized Raman spectroscopy in the study of some SAPA architectures.

### 3.2. Polarized Imaging and Birefringence Monitoring

Another optical polarization-based measurement method that is used for studying peptides’ and amino acids’ micro- and nanostructures is polarized optical microscopy (POM) [50,51]. Depending on the setup and components, one can obtain quantitative information with respect to the SAPA architectures (such as the 16 elements of a Mueller matrix [52], retardation, refractive index, thickness, and birefringence [50]) and also polarization imaging information.

Simple POM is based on the common brightfield microscope with additional components [50,52]. The additional components usually include a (rotatable) PSG (used to polarize the incident light), strain-free microscope objectives (used to reduce unwanted birefringence of the objective’s lens), and an analyzer that is rotated at 90° with respect to the polarizer (a polarizer is an optical element that generates a specific type of polarized light (linear, circular, or elliptical) [38]. The analyzer (also known as a PSA or polarization state detector—PSD) is just another polarizer placed after the specimen.

A further component that can be added to a POM setup is a compensator (also known as a retarder). A retarder is an optical device that creates a phase shift between two orthogonal components of polarized light. If the retarder is a quarter-wave plate, circular polarization is produced by the linear polarization.

The PSA component can be replaced by a polarization camera that is composed of a complementary metal-oxide semiconductor (CMOS) or a charge-coupled device (CCD) sensor and a grid of polarizer arrays arranged at the 0°, 45°, −45°, and 90° transmission axes. A major advantage of using a polarization camera as a PSA is that it allows the easy and rapid extraction of the intensity, azimuth of linear polarization (AoLP), and degree of linear polarization (DoLP). From these triplet parameters, the 12 linear elements of the Mueller matrix that characterize the specimen can be deduced [53]. In the following paragraphs, some examples of the use of POM and a polarization camera to study amino acids and peptides are given.

In the study of amino acids, a polarization camera was deployed by Ellis et al. [54] in order to determine the L- and D- enantiomeric abundances of the Serine (Ser, S) and phenylalanine (Phe, F) amino acids. This was performed by computing their optical rotation (which is the rotation angle of a plane-polarized light after passing through a molecule) as a function of concentration. The polarization camera yielded the AoLP, and, from it, the optical rotation was calculated [54]. Note that the AoLP is related to the Stokes vector by $\frac{1}{2}\tan^{-1}\left(S_{2}/S_{1}\right)$ [51]. Ellis et al. found a lower bound on the amino acid concentration, above which their optical rotation can be detected [54]. Such method to detect small amounts of amino acids is of great importance, for example, in extra-terrestrial biosignature research [54].

Another amino acid that was studied using POM is Histidine (His, H). Histidine (His, H) is a polar hydrophilic α-amino acid that contains an imidazole side chain and is capable of self-assembly into two polymorph crystal structures—monoclinic (P2_1_) and orthorhombic (P2_1_2_1_P_1_) [55]. The interaction of linear polarized light with self-assembled Histidine microplates (with an orthorhombic crystal structure) was studied by Handelman et al. using POM that included a polarization camera [56]. Using this setup, the triplet parameters (intensity, DoLP, and AoLP) at the output (camera) plane were extracted as a function of PSG rotation angle. Note that the DoLP is related to the Stokes vector by $\mathrm{DoLP}=\sqrt{S_{1}^{2}+S_{2}^{2}}/S_{0}$ [51].

**Figure 3:**
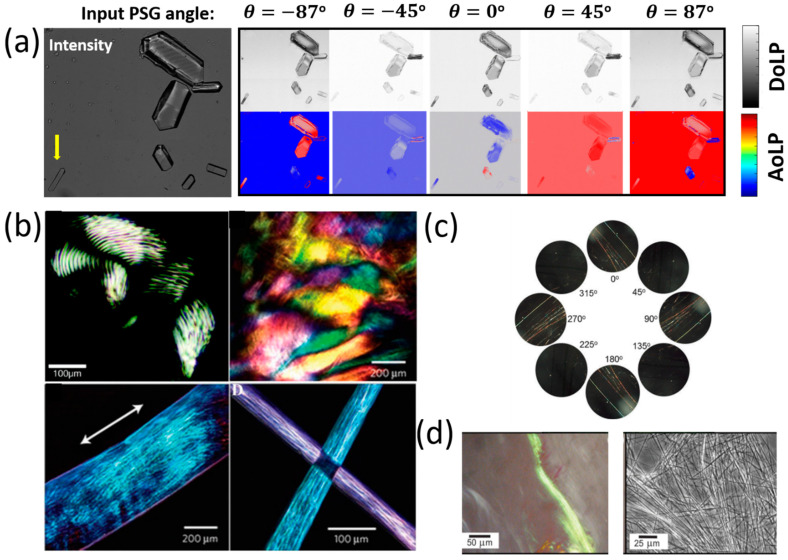
Polarized imaging microscopy of peptides and amino acids. (**a**) Intensity, DoLP, and AoLP images of seven different Histidine microstructures as a function of input PSG angle [56], reproduced with permission from A. Handelman, et al., *Adv. Func. Mat*, published by John Wiley & Sons, 2020. (**b**) Polarized light microscopy images of peptide amphiphile (PA) gels: (**top-row**, **left**) PA gel, formed by short anisotropic domains; (**top-row**, **right**) PA gel film, formed by noodle-like gels; (**bottom-row**, **left**) one noodle-like PA gel string shows aligned monodomain (**bottom-row**, **right**); light extinction at cross-points of two PA gel strings shows uniform alignment in each string [34], reproduced with permission from Rashad Mammadov, Ayse B. Tekinay, Aykutlu Dana, Mustafa O. Gule., *Micron*, published by Elsevier, 2012. (**c**) Cross-polarized microscopy images of diphenylalanine (FF) microstructures assembly at different angles after one cryogenic treatment [58], reproduced with permission from Junbai Li, Pengli Zhu, Wei Cui, et al., *Angewandte Chemie International Edition*, published by John Wiley & Sons, 2017. (**d**) Birefringence observed by polarized optical microscopy upon staining fibrillar superstructure of peptide amphiphile with Congo red [59], reproduced with permission from I. W. Hamley, *Chem. Commun.*, published by RSC, 2015.

Figure 3a shows the DoLP, AoLP, and intensity images of several His-microplates at different sizes, thicknesses, and orientations at five PSG angles (−87°, −45°, 0°, 45°, 87°). The different colors of the His-microplates that are seen in the DoLP and AoLP images result from the different thicknesses and orientations of the His-microplates. Further, in that study [56], the birefringence of the His-microplates was extracted by elimination of the corresponding thickness and orientation values of the His-microplates and by considering their optical symmetry (biaxial) and crystal system (orthorhombic).

Besides amino acids, self-assembled peptide microstructures were also investigated by POM methods [34] (Figure 3b). For example, Stupp et al. [57] developed a method (based on thermal treatment) for the fabrication of long arrays of aligned peptide nanofibers bundles. In order to evaluate their method, Stupp et al. used POM in order to image the birefringence of these peptide amphiphile gel nanofibers. It was shown that the fabrication using thermal treating presented in [57] yields macroscopic birefringent domains of the order of tenths of millimeters.

POM was also used to image the birefringence of SAPA micro- and nanostructures. For example, Li et al. [58] developed a cryogenic-treatment-based technique to control the self-assembly of dipeptide diphenylalanine (FF) microstructures (Figure 3c). Using POM, Li et al. found that birefringence was strong and angle-dependent after the cryogenic treatment, which proved the feasibility of their method to form well-ordered, chiral crystalline dipeptide fibers from their organogel phase.

Some works use POM to image peptide-fibrils that were stained with organic dyes (such as Congo red) in order to detect amyloid formation by imaging the peptides’ birefringence. Such works are, for example, [59,60], where (in [59]) birefringence was detected in stained lipopeptides fibrils (Figure 3d), and (in [60]) birefringence was imaged in stained (*N*-fluorenylmethoxycarbonyl) Fmoc-RGD peptide hydrogels.

POM was also used to monitor the birefringence of peptides as a function of time and temperature. This monitoring allows for the examination of the effect of external conditions on the self-assembly process of the peptides’ micro- and nanostructures.

**Figure 4:**
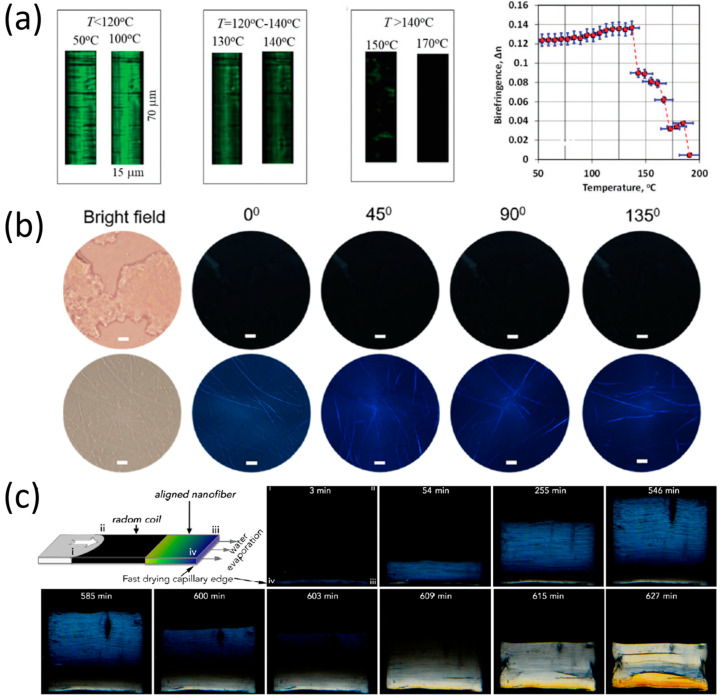
Monitoring birefringence in peptide micro- and nanostructures. (**a**) Evolution of birefringence of triphenylalanine (FFF)-microplates under helical to β-sheet thermally induced refolding [61], reproduced with permission from Boris Apter, Nadezda Lapshina, Igor Lapsker, et al., *Advanced Optical Materials*, published by John Wiley & Sons, 2021. (**b**) Cross-polarized microscopy images of Fmoc-FF assemblies obtained at 1 min (first row) and 3 min (second row) sonication time [62], reproduced with permission from Jingwen Song, Ruirui Xing, Tifeng Jiao, et al., *ACS App. Mat. Inter*., published by American Chemical Society, 2018. (**c**) Monitoring the formation of birefringence lamellar domains from naphthalene-FF solution by polarized optical microscopy [63], reproduced with permission from Shijin Zhang, William Cortes, Toshio Sasaki, et al., *ACS Applied Bio Materials.*, published by American Chemical Society, 2020.

For example, Rosenman, Apter et al. studied the evolution of the birefringence of tri-phenylalanine (FFF) tripeptide microplates as a function of temperature, using POM [61]. It was shown that birefringence decreases substantially as the heating temperature of the FFF-microplates rises (Figure 4a). Their conclusion, from the decrease in birefringence of heated FFF microplates, was that thermally induced FFF-microplates exhibit a transformation from a helix to a β-sheet secondary structure, which correlates with the circular dichroism (CD) spectra observations of such a transformation [61]. A further discovery made using POM was that the FFF-microplates undergo an intermediate melt-like state before they complete their full transformation into a β-sheet secondary structure.

In addition to the temperature-dependent birefringence discussed above, time-dependent birefringence was measured by Yan et al. in other peptide nanostructures (such as Fmoc-FF) in order to track their formation process [62]. The transformation of Fmoc-FF triclinic aggregates to nanofibers and to monoclinic nanobelts was initiated by ultrasound irradiation and monitored by POM imaging. Figure 4b shows the polarization images of Fmoc-FF aggregates after a 1 min ultrasound sonication (first row) and polarization images of Fmoc-FF nanofibers after a 3 min ultrasound sonication (second row). It can be noted that no obvious anisotropic birefringence was observed after the 1 min sonication of the Fmoc-FF aggregates, but strong birefringence was observed after the 3 min sonication of the Fmoc-FF nanofibers. This clearly shows the ultrasound-dependent evolution of the nanofibers from Fmoc-FF aggregates.

POM was also used to monitor the transformations of disordered peptide structures into highly ordered crystalline structures. Zhang et al. introduced a method (based on the differential evaporation rates of peptide solution) of uniformly aligning naphthalene-FF (Nap-FF) peptide nanofibrils [63]. POM was used in this work to track the orientation transition of these nanofibers in real time. Figure 4c depicts images (acquired by POM) of the casting of a Nap-FF based solution after various time periods of evaporation. As can be seen in Figure 4c, the evolution of the self-assembly of peptide-nanofibrils can be divided into several time intervals. Within the first 540 min, the birefringence area increases, suggesting the condensation of the peptide nanofibrils. In the next 60 min, the birefringence area shrinks, and the nanofibrils accelerate their solidification, showing a white interference color [63]. After the completion of solidification, four birefringence lamellar domains are formed. This formation can be seen in the different colors of the domains in the POM image.

The examples mentioned in this section show that POM has been used to monitor the self-assembly of peptide nanostructures, derive the birefringence of SAPA microstructures, track changes in the secondary structures of peptides, and verify the feasibility of various peptide structures’ fabrication processes.

### 3.3. Polarization and Fluorescence

Fluorescence is the emission of light as a result of molecular excitation by light absorption [64]. If the excited light is polarized, the absorption of the fluorophore is proportional to $\cos^{2}\theta$, where θ is the angle between the electric field vector of the excited light and the absorption transition moment vector. This means that when θ = 90°, i.e., the polarized electric field vector is oriented at 90° in relation to the orientation of the transition dipole moment of the molecules [65], then the probability of excitation will be minimal. When the polarized electric field vector is aligned (i.e., parallel) with the transition dipole moment of the molecules, then the probability of excitation will be maximal. As such, polarization-based fluorescence measurement tools can be used to study the molecular organization of fluorophores [64] and the effect of the chemical environment on the fluorophore.

Common polarization-based fluorescence measurement methods include Fluorescence Polarization Microscopy (FPM), Muller Fluorescence Spectroscopy (MFS), and Circularly Polarized Luminescence (CPL) Spectroscopy. These instruments are widely used in life-science applications [65], for example, for the study of protein structures [66,67] and disease diagnostics [68]. FPM and MFS measurements can also be used in cases where fluorescent dyes (such as Thioflavine-T and Congo Red) are incorporated with non-fluorescent molecules [69]. In the following paragraphs, some recent examples of the use of these instruments for the characterization of SAPA micro- and nanostructures are provided.

**Figure 5:**
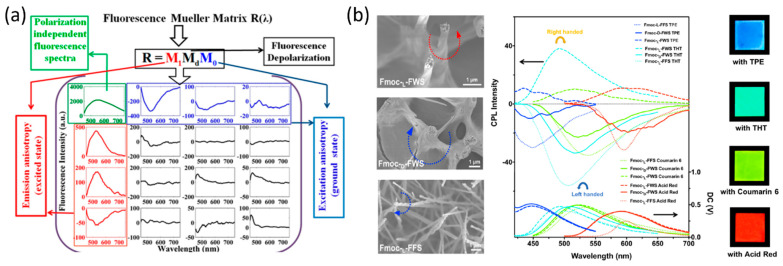
(**a**) 4 × 4 fluorescence spectroscopic Mueller matrix of painted peptides nanotube [70], reproduced with permission from Krishnendu Maji, Sudipta Saha, Rajib Dey, et al., *J. Phys. Chem. C*, published by ACS, 2017. (**b**) Co-assembly of the Fmoc-tripeptides with the various achiral fluorescent molecules and their CPL spectra [71], reproduced with permission from Qing Li, Jiaxing Zhang, Yuefei Wang, et al., *Nano Letters*, published by ACS, 2021.

Haldar et al. used MFS to probe the anisotropic molecular organization and orientation of Boc-Xaa-Met-OMe (Xaa = Val/Leu) peptide nanotubes painted by the organic dye 2,3,6,7-tetrabromonaphthalene diimide (TB-NDI) [70]. The full 4 × 4 fluorescence spectroscopic Mueller matrix (Figure 5a) was derived, and, by performing inverse analysis, Haldar et al. were able to quantify the fluorescence linear diattenuation, the linear polarizance, and the average fluorescent dipolar orientation angles for the ground and excited molecular states [70]. Eventually, these parameters were used to determine the molecular angular distribution function and the molecular orientational order.

He et al. developed a method of generating CPL with inverted handedness from a Fmoc-tripeptides film [71]. He et al. used a CPL Spectrometer in order to show that, by changing the middle amino acid (Phe and Trp) of Fmoc-tripeptides, and with the addition of achiral fluorescent dyes, CPL emission was observed after the peptides self-assembled into long-range-ordered hierarchical helical arrays (Figure 5b). The generation of CPL from peptide microstructures extends the diversity of optical materials that are able to generate CPL, a feature that is used for bioimaging [72], optical devices [73], and chirality transfer and energy transfer studies [74].

The examples mentioned in this section show that polarization-based fluorescence measurement tools have been used to derive the anisotropic molecular organization of peptides and to test peptides nanostructures CPL capability.

## 4. Polarized Waveguiding in Self-Assembled Amino Acid Microstructures

Bio-organic optical waveguides show a great potential in various biomedical applications, such as photodynamic therapy [75], photobiomodulation [76,77], and bioresorbable photonics [78]. Polarized waveguiding at the microscale is also of great importance for a variety of photonic applications, such as for increasing the polarization efficiency of liquid crystal displays [79,80] and for polarized data decoding [81]. Thus, major research efforts are aimed at finding more organic materials that can guide light at various scale sizes [82].

One study that recently demonstrated polarized optical waveguiding in organic SAPA microstructures is described in [56]. In that work, Handelman et al. showed the ability of an amino acid Histidine irregular convex hexagonal (His-ICH) microstructure to passively guide linear polarized light [56].

**Figure 6:**
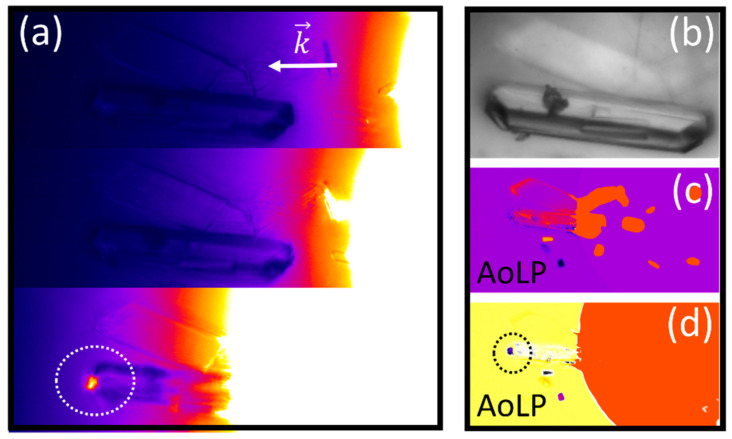
Passive polarized waveguiding in Histidine irregular convex hexagonal (ICH) microstructure. (**a**) Intensity image of passive optical waveguiding, (**b**) brightfield microscopy imaging of the single Histidine ICH microstructure, (**c**) image of the azimuth of linear polarization at input polarization angle $\theta =7^{o}$, and (**d**) image of the azimuth of linear polarization at input polarization angle $\theta =-60^{o}.$

Figure 6 depicts passive (polarized) waveguiding in His-ICH microstructure [56]. The passive waveguiding capability can be clearly seen in Figure 6a, where, only when light impinges upon the His-ICH microstructure (presented in Figure 6b) at a specific location, a small light spot appears at the opposite end of the microstructure (marked by a white circle). However, when light impinges upon the His-ICH microstructure at other locations, no light spots can be observed at the end of the microstructure.

The capability of guiding *polarized* light is evident from the azimuth of linear polarization (AoLP) images presented in Figure 6c,d. Only at a specific input polarization state, a light spot at the opposite end of the His-ICH microstructure is observed (Figure 6d). This proves that His-ICH plates can guide polarized light.

## 5. Final Remarks and Future Directions

**Figure 7:**
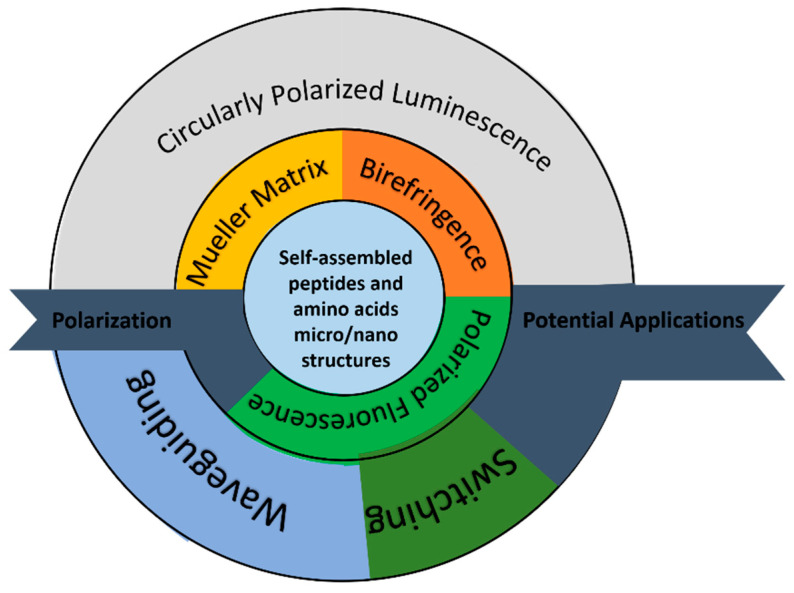
Infographics diagram that summarizes the optical applications and the parameters that were extracted from the polarization-based measurement methods discussed in this paper.

The great interest in SAPA micro- and nanostructures has accelerated the use of various optical measurement methods for analyzing their physical and chemical properties. In this review, it was shown that polarization-based optical measurement methods can deduce additional information regarding the inner structures of SAPA. Examples of the specific information obtained by polarization-based measurement methods and described in this review are birefringence, secondary-structure tracking, molecular orientation, and the monitoring of the peptide self-assembly processes. These physical parameters may be useful for future SAPA-based photonic applications. An example of a property that is discussed in this paper and was discovered using one such polarization-based measurement method is polarized optical waveguiding in a microstructure of the amino acid Histidine. This polarized optical waveguiding capability has the potential for light-guiding applications within or between organic elements. Figure 7 shows an infographic diagram that summarizes the optical applications and the parameters that were extracted from the polarization-based measurement methods discussed in this paper.

There are many future directions that can be taken in this research field. For example, other SAPA-based microstructures can be explored in the context of linear or circular polarization waveguiding. Furthermore, polarized fluorescence at various wavelengths can be investigated in other SAPA structures. Precise birefringence studies, including monitoring changes in birefringence as a function of external parameters such as temperature, evaporation rate, and pH environment, can also be performed for many types of SAPA crystals. It is, therefore, recommended to expand the implementation of these polarization-based measurement methods for further SAPA research.
